# The *Thoc1* Encoded Ribonucleoprotein Is Required for Myeloid Progenitor Cell Homeostasis in the Adult Mouse

**DOI:** 10.1371/journal.pone.0097628

**Published:** 2014-05-15

**Authors:** Laura Pitzonka, Sumana Ullas, Meenalakshmi Chinnam, Benjamin J. Povinelli, Daniel T. Fisher, Michelle Golding, Michelle M. Appenheimer, Michael J. Nemeth, Sharon Evans, David W. Goodrich

**Affiliations:** 1 Department of Pharmacology & Therapeutics, Roswell Park Cancer Institute, Buffalo, New York, United States of America; 2 Department of Immunology, Roswell Park Cancer Institute, Buffalo, New York, United States of America; University of Toronto, Canada

## Abstract

Co-transcriptionally assembled ribonucleoprotein (RNP) complexes are critical for RNA processing and nuclear export. RNPs have been hypothesized to contribute to the regulation of coordinated gene expression, and defects in RNP biogenesis contribute to genome instability and disease. Despite the large number of RNPs and the importance of the molecular processes they mediate, the requirements for individual RNP complexes in mammalian development and tissue homeostasis are not well characterized. THO is an evolutionarily conserved, nuclear RNP complex that physically links nascent transcripts with the nuclear export apparatus. THO is essential for early mouse embryonic development, limiting characterization of the requirements for THO in adult tissues. To address this shortcoming, a mouse strain has been generated allowing inducible deletion of the *Thoc1* gene which encodes an essential protein subunit of THO. Bone marrow reconstitution was used to generate mice in which *Thoc1* deletion could be induced specifically in the hematopoietic system. We find that granulocyte macrophage progenitors have a cell autonomous requirement for *Thoc1* to maintain cell growth and viability. Lymphoid lineages are not detectably affected by *Thoc1* loss under the homeostatic conditions tested. Myeloid lineages may be more sensitive to *Thoc1* loss due to their relatively high rate of proliferation and turnover.

## Introduction

The co-transcriptional packaging of nascent RNA transcripts into RNP complexes is important for transcription, RNA processing, and RNA export from the nucleus [1]. RNP complexes are composed of multiple protein and RNA subunits. They are heterogeneous and dynamic, differing in composition depending on the transcript and the stage of the transcript’s life cycle. Potential combinatorial permutations are vast enough to facilitate unique RNP processing pathways for different subsets of transcripts [2]. These observations have inspired the hypothesis that co- and post-transcriptional RNP mediated mechanisms support the elaboration of coordinated gene expression [3], [4]. Loss of RNP function can also compromise genome integrity directly by inducing promiscuous formation of R-loops, a three-strand nucleic acid structure composed of an RNA∶DNA hybrid formed during transcription plus a displaced DNA strand [5]. R-loops lead to DNA strand breaks by multiple mechanisms. While the importance of RNP mediated mechanisms for gene expression and genome integrity is increasingly appreciated, the contribution of individual RNP complexes to normal growth and development in mammals is not well characterized. Identifying these contributions will further the understanding of how defects in RNP mediated processes lead to disease [6].

THO is an RNP complex that assembles on nascent RNA in a RNA cap, splicing, and ATP-dependent manner [7], [8]. It recruits RNA export factors to form larger complexes like TREX that facilitate interaction with and activation of the nuclear export apparatus [9]–[11]. THO, therefore, physically links transcription with nuclear export [12]–[16]. In addition to defects in nuclear export, THO deficiency affects other steps in transcription that depend on proper RNP biogenesis including transcriptional elongation [17]–[19], transcription-associated recombination [20]–[22], and transcript 3′ end formation [23]. THO is conserved from yeast to man. In metazoa, THO is composed of six proteins in equal stoichiometry encoded by the *THOC1, THOC2*, *THOC5*, *THOC6, THOC7*, and *Tex1* genes [15]. The yeast THO orthologue contains four proteins encoded by the *THO2*, *HPR1*, *MFT1*, and *THP2* genes [12]. Loss of any one of these yeast proteins causes disassembly of the complex, loss of function, and the same set of deficiency phenotypes. Surprisingly, given its widespread role in RNA processing and transport [20], THO is not essential for yeast viability.

Deletion of *Thoc1* or *Thoc5* in mice yields an embryonic lethal phenotype [24], [25]. Since THO is required for early embryonic development, testing the requirements for THO in maintaining homeostasis in adult tissues has been limited. To overcome this limitation, a floxed mouse allele has been generated allowing inducible deletion of the *Thoc1* [26]. Using this allele, widespread *Thoc1* deletion has been observed to cause cell type specific effects such as disruption of stem cell homeostasis in small intestine but not the related mucosa of the large intestine [27]. This suggests *Thoc1* deficiency has context dependent effects. Here the effects of *Thoc1* deletion on hematopoiesis are examined.

## Materials and Methods

### Mice, Tamoxifen Treatment, and Tissue Harvest

All animal work was approved by the Roswell Park Cancer Institute Animal Care and Use Committee according to AAALAC standards. The generation and genotyping of the *Rosa26Cre^ERT2^* and floxed *Thoc1* alleles were described previously [26], [28]. Widespread *Thoc1* deletion was performed as previously described [27]. Mice successfully reconstituted with *Thoc1^f/f^Rosa26Cre^ERT2/+^* test or *Rosa26Cre^ERT2/+^* control bone marrow were treated with five daily injections of 2 mg tamoxifen in corn oil at 9 to 10 weeks post transplantation via intraperitoneal injection. Following tamoxifen administration, mice were euthanized by CO_2_ inhalation and tissues collected as previously described [27]. Tissue was either snap frozen for later extraction of RNA and protein or fixed and embedded to generate tissue sections.

### Blood Cell Counts, Blood Smears, and Bone Marrow Smears

20–50 µl of peripheral blood was collected retro-orbitally into EDTA containing tubes and complete blood counts performed using an MS4-5 Automated Hematology Cell Counter (Melet Schloesing Labratories). The total numbers of white blood cells, red blood cells, and platelets, as well as the percent of lymphocytes, neutrophils, monocytes, eosinophils, and basophils within the white blood cell population were counted. Peripheral blood smears were prepared from retro-orbital bleeds, fixed in methanol, and stained with Wright-Giemsa stain. Bone marrow was extracted from the right femur, cleaned of muscle and fat tissue, and was halved length wise. Sable hair (3/O) brush and 5% BSA in 1X PBS was used to brush bone marrow cells on to microscope slides and the slides were then fixed in methanol and stained with Wright-Giemsa. For each time point, individual mice were used so that blood was not collected from the same mouse more than once.

### Bone Marrow Reconstitution and Flow Cytometry

Bone marrow chimeric mice were generated as described [29]. Briefly, *Rosa26Cre^ERT2/+^* and *Thoc1^f/f^Rosa26Cre^ERT2/+^* 8-week old mice on the C57BL/6 background (expressing CD45.2) were sacrificed and their bone marrow transplanted into sub-lethally irradiated, age and sex-matched B6.SJL-*Ptprc* ^a^/BoyAiTac mice that express CD45.1 (Taconic laboratories). Bone marrow was extracted from both femurs and tibias by flushing with sterile PBS using a 27 gauge needle and stored on ice. 1×10^6^ bone marrow cells in 200 µl PBS were transplanted into recipient mice via tail vein injection. Prior to transplantation, recipient mice were irradiated twice at a 3 hour interval with 5 Gy over the course of 2.4 minutes. All irradiated recipient mice were maintained on the antibiotics sulfamethoxaole and trimethoprim (Bactrim(R), Hi-Tech Pharmacal, Amityville, NY) administered orally via in the drinking water.

Bone marrow reconstitution in recipient mice was monitored by immunophenotyping of peripheral blood. Retro-orbital blood was collected in EDTA tubes at 4 and 9 weeks post transplantation, red blood cells lysed with ACK lysis buffer (150 µM NH4Cl, 9.9 mM KHCO3, 120 µM EDTA), and the CD45.1 and CD45.2 markers immunostained with 0.5 mg/ml PE conjugated antibody (BD Pharmingen, #550802) and 0.2 mg/ml FITC-conjugated antibody (BD Pharmingen #560695). Samples were analyzed on an LSR II Flow Cytometer (BD Life Sciences). The ratio of CD45.2 to CD45.1 positive cells from at least 50,000 events estimates the extent of bone marrow reconstitution.

Immunophenotyping of different cell lineages in the bone marrow, peripheral blood, lymph node, and spleen was performed similarly. Bone marrow samples were treated with ACK lysis buffer as above and 5×10^6^ remaining bone marrow cells re-suspended in 100 µl staining buffer (.5% FBS in PBS) with 1 µl of the appropriate antibody. For analysis of peripheral blood, serum was collected and double lysed in ACK lysis buffer before staining. Spleen cells were obtained by physical dissociation, filtering, and ACK lysis before staining. Lymph node cells were obtained similarly without ACK lysis. After incubation on ice, cells were washed with staining buffer and resuspended in staining buffer plus DAPI prior to flow cytometry analysis. Samples, along with unstained or DAPI only stained cells were analyzed on a LSRII flow cytometer. Data was analyzed with Flowjo v9.5 software (TreeStar). Live singlets were gated on DAPI- as well as side scatter and forward scatter singlets in order to eliminate false positive events. Cells were analyzed for markers that can be used to identify distinct hematopoietic stem/progenitor cell populations including Lin (CD4, CD8, B220, Ter-119, Gr-1, CD11b), Sca-1 (D7), C-kit (C2B8), CD150 (mshad), CD105 (Mu7/18), and CD16/32 (93). All antibodies were obtained from eBioscience. The antigens and the corresponding progenitor populations they define are listed in Table S1.

For analysis of the cell cycle, cells were stained with the cell surface markers above and then fixed 10 minutes at room temperature with 2% paraformaldehyde. Cells were washed with 0.5% saponin, 3.0% FBS in PBS and resuspended with 20 µg/ml Hoescht 33343 (Sigma Aldrich) for 30 minutes at room temperature. After washing and resuspension in PBS, cells were analyzed by flow cytometry. Cell cycle phases were determined by DNA content as measured by Hoechst 33343 staining intensity. Flowjo software v10.0 (TreeStar) was used for cell cycle analysis of flow data.

### Colony Formation Assays and *In vitro* Assays

40,000 viable bone marrow cells per recipient mouse were washed with 1x PBS and re-suspended in 200 µL sterile 1X PBS. The cells were added to 4 mL of methylcellulose-based medium with recombinant cytokines and EPO for mouse cells (Methocult GF #M3434, StemCell Technologies, Vancouver, BC) containing Pen/Strep (Invitrogen, Grand Island, NY). The bone marrow methylcellulose media mixture was plated and incubated at 37°C. Total numbers of colonies were counted on day 7.

For isolation of pre-granulocyte-macrophage progenitor (pre-GMP) and granulocyte-macrophage progenitor (GMP) populations, bone marrow was harvested and pooled from a total of seven mice each with the *Thoc1^F/F^*:*Rosa26^CreERT2^* or *Thoc1^+/+^*:*Rosa26^CreERT2^* genotype in two independent experiments. Bone marrow cells were lineage depleted by incubating with the following rat anti mouse lineage antibodies: CD4 (clone GK1.5), CD8a (53-6.7), B220 (RA3-6B2), Gr-1 (RB6-8C5), Mac-1 (M1/70), Ter119 (Ter-119). After 15 minutes of incubation on ice, cells were washed and incubated for an additional 15 minutes on ice with anti-rat antibody magnetic beads (Qiagen). Unbound lineage-negative cells were harvested and stained for the aforementioned GMP markers using rat anti-mouse Sca-1 (D7), c-kit (2B8), CD150 (mShad), CD105 (MJ7/18), and CD16/32 (93) (Table S1). Phenotypic definitions of pre-GMP and GMP populations were based on those described in Pronk, *et al.* [30]. Sorted GMPs were cultured in serum-free StemSpan medium (Stem Cell Technologies) with 25 ng/ml recombinant mouse SCF, FLT-3 and GM-CSF (Peprotech). Tamoxifen (2 µM) or ethanol vehicle was added to the cultures on day 0 and day 1, and then washed out on day 2. On day 4, cells where harvested and stained with antibodies directed against the mature granulocyte/macrophage markers Gr-1 and Mac-1. Cells were then washed and resuspended in binding buffer with Annexin V according to manufacturer’s recommendations (eBioscience). After washing, cells were resuspended with DAPI to discriminate between live and dead cells. GMPs were defined as cells negative for Gr-1 and Mac-1

## Results

### Widespread *Thoc1* Deletion in Adult Mice Affects Hematopoiesis

**Figure 1:**
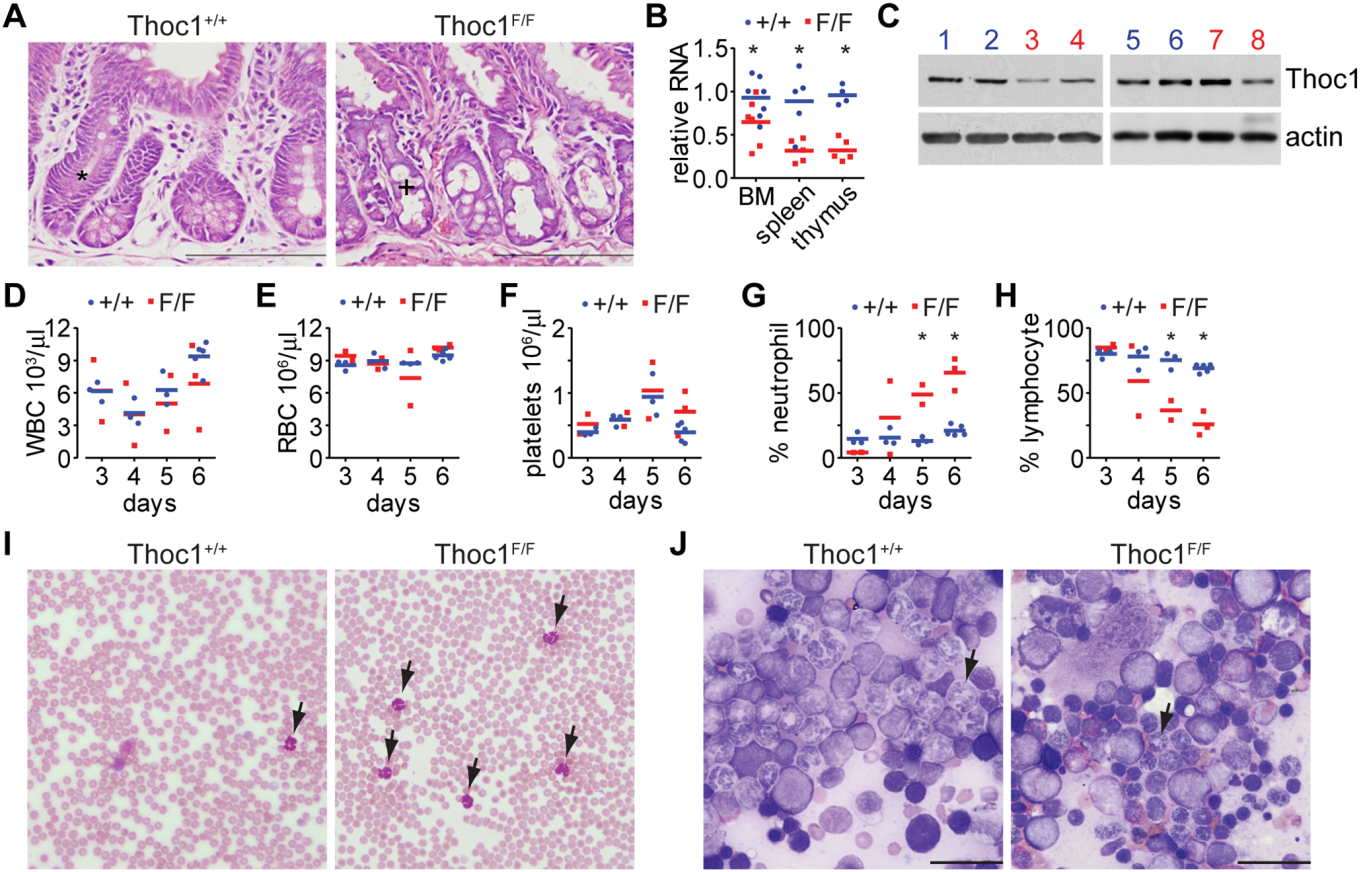
Widespread *Thoc1* deletion affects hematopoiesis. A) The small intestine from *Thoc1^F/F^*:*Rosa26^CreERT2^* (Thoc1^F/F^) or *Thoc1^+/+^*:*Rosa26^CreERT2^* (Thoc1^+/+^) mice was dissected six days from to the start of tamoxifen treatment, tissue sections were stained with H&E, and representative images are shown. The asterisk identifies a typical normal small intestinal crypt. The plus sign identifies a degenerating, hypocellular crypt. Scale bars represent 200 microns. B) Bone marrow (BM), spleen, and thymus tissue was isolated from Thoc1^F/F^ (red) or Thoc1^+/+^ (blue) mice 5 days after the start of tamoxifen treatment. RNA was extracted, and the fraction of remaining wild type *Thoc1* RNA quantitated by real time RT-PCR. Each data point is from a different mouse, and the data are normalized to one of the control mice for each tissue. Significant differences between genotypes (t-test P<0.05) are indicated by asterisks. C) Protein extracted from thymus (1–4) or spleen (5–8) of Thoc1^F/F^ (red) or Thoc1^+/+^ (blue) mice 6 days from the start of tamoxifen treatment was analyzed for *Thoc1* protein by western blotting. Each lane represents an individual mouse. Actin serves as the loading control. D) *Thoc1^F/F^*:*Rosa26^CreERT2^* (red) or *Thoc1^+/+^*:*Rosa26^CreERT2^* (blue) mice were treated with tamoxifen, peripheral blood was isolated, and white blood cells (WBC) were counted at the indicated times from the start of treatment. Each data point is from a different mouse of the indicated genotype. E) Peripheral red blood cells (RBC) from mice in D) were counted. F) Peripheral blood platelets from mice in D) were counted. G) The percentage of neutrophils among total peripheral white blood cells from mice in D) was measured. Each data point is from a different mouse. Significant differences between genotypes (t-test P<0.01) are indicated by asterisks. H) The percentage of lymphocytes among total peripheral white blood cells was counted as in G. I) Peripheral blood was collected from tamoxifen treated *Thoc1^F/F^*:*Rosa26^CreERT2^* (*Thoc1^F/F^*) or *Thoc1^+/+^*:*Rosa26^CreERT2^* (*Thoc1^+/+^*) mice, and Giemsa stained blood smears were examined under light microscopy. Representative images are shown with neutrophils highlighted by arrows. J) Bone marrow brush smears from tamoxifen treated *Thoc1^F/F^*:*Rosa26^CreERT2^* (*Thoc1^F/F^*) or *Thoc1^+/+^*:*Rosa26^CreERT2^* (*Thoc1^+/+^*) mice were stained with Wright Giemsa and imaged under light microscopy. Representative images are shown with neutrophils typical of each sample highlighted by arrows. The scale bar represents 50 microns.

A floxed allele of *Thoc1* in which loxP sites flank exons 6–7 was previously created (*Thoc1^F^*), and Cre mediated deletion creates the equivalent of a null allele [26]. *Thoc1^F^* alleles were bred into mice containing a transgene expressing the Cre recombinase-estrogen receptor fusion protein (*Rosa26^CreERT2^*) to allow widespread, tamoxifen inducible *Thoc1* gene deletion in adult mice [27]. Untreated and thus undeleted *Thoc1^F/F^*:*Rosa26^CreERT2^* mice were viable and did not exhibit phenotypes distinguishable from wild type mice. Nine to twelve week old *Thoc1^F/F^*:*Rosa26^CreERT2^* test mice or *Thoc1^+/+^*:*Rosa26^CreERT2^* control mice were administered tamoxifen daily for 5 days to drive widespread Cre mediated *Thoc1* gene deletion. As previously described, tamoxifen treatment caused decreased stem/progenitor cell proliferation and viability in the small intestinal crypt [27]. This defect compromised the structural integrity of the small intestine (Fig. 1A) causing death beginning at 11 days from the start of tamoxifen treatment. The small intestine has one of the highest rates of cell turnover during homeostasis, suggesting rapidly proliferating cell types are particularly susceptible to *Thoc1* deficiency. This prompted us to explore whether *Thoc1* loss had effects on the hematopoietic system, another tissue characterized by rapid cell turnover.

Wild-type *Thoc1* RNA levels were significantly reduced in the bone marrow, spleen, and thymus of tamoxifen treated *Thoc1^F/F^*:*Rosa26^CreERT2^* test mice (Fig. 1B) indicating induced deletion of floxed *Thoc1* alleles was efficient in these tissues. *Thoc1* protein levels were also generally reduced (Fig. 1C), but the effects in individual mice were variable. Despite *Thoc1* deficiency in these hematopoietic tissues, the number of white blood cells, red blood cells and platelets in peripheral blood was not significantly different from control mice for the time points measured (Fig. 1D–F). The relative proportion of neutrophils was significantly increased whereas the fraction of lymphocytes declined in tamoxifen treated *Thoc1^F/F^*:*Rosa26^CreERT2^* test mice compared to tamoxifen treated *Thoc1^+/+^*:*Rosa26^CreERT2^* control mice (Fig. 1G,H). The relative increase in neutrophils was confirmed by examination of peripheral blood smears (Fig. 1I). Brush smears suggested that the bone marrow from *Thoc1* deficient test mice was abnormal (Fig. 1J). For example, bone marrow neutrophils from tamoxifen treated *Thoc1^F/F^*:*Rosa26^CreERT2^* mice appeared smaller with more condensed nuclei. These observations suggested that *Thoc1* deficiency affects hematopoiesis. Because widespread *Thoc1* deficiency affected the small intestine, however, non-cell autonomous effects might influence the hematopoietic phenotype observed. In particular, loss of small intestine structural integrity would be expected to cause inflammatory and immune responses that may influence mobilization of neutrophils from the bone marrow into the peripheral blood [27].

### Effects of *Thoc1* Deletion in Bone Marrow Reconstituted Mice

**Figure 2:**
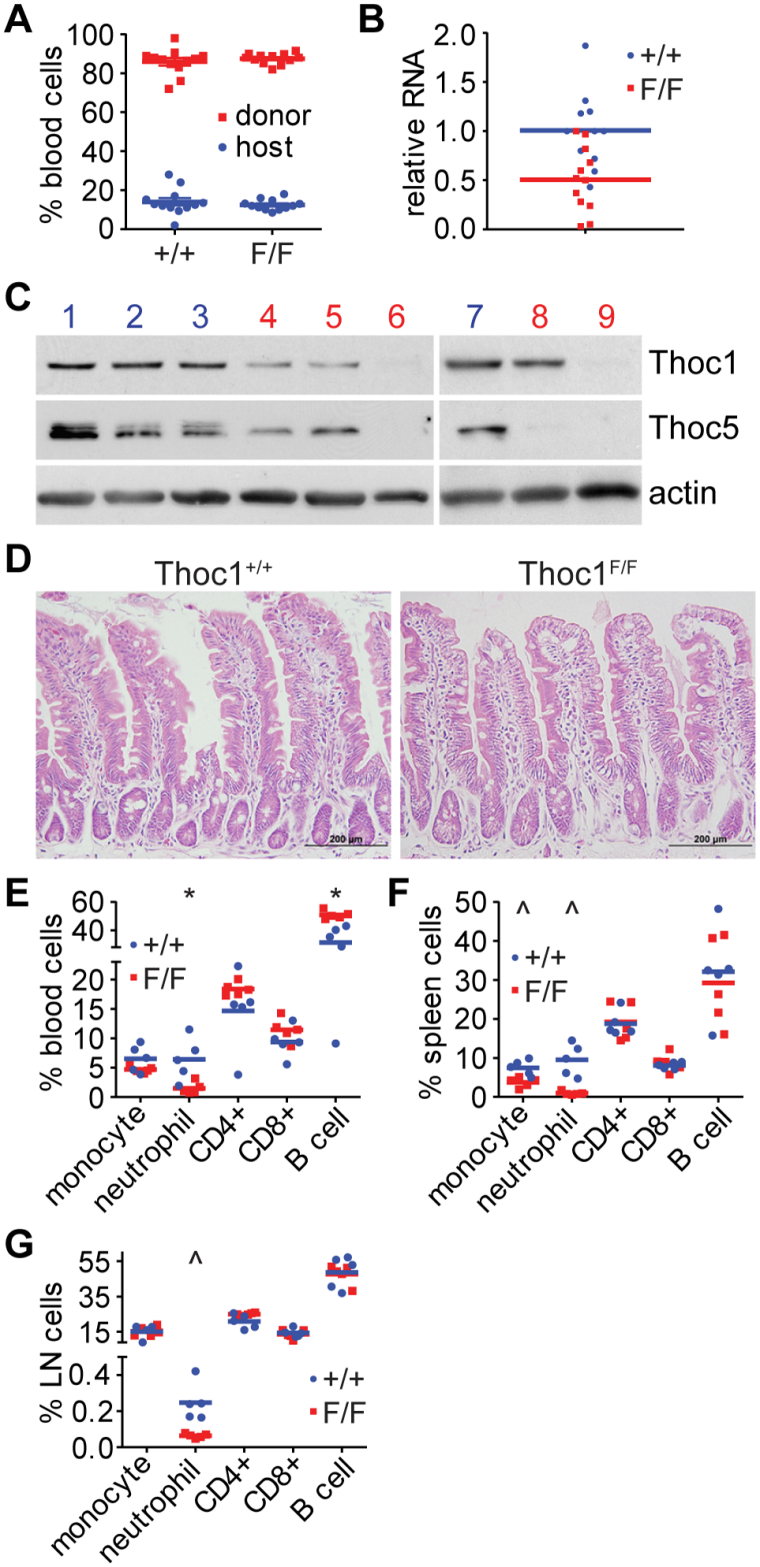
Thoc1 deficiency in the bone marrow causes a decline in myeloid cells, but not lymphocytes. A) CD45.1 wild type mice were irradiated and transplanted with bone marrow from Cd45.2 *Thoc1^F/F^*:*Rosa26^CreERT2^* (*Thoc1^F/F^*) or *Thoc1^+/+^*:*Rosa26^CreERT2^* (*Thoc1^+/+^*) mice. 9 weeks later, CD45.1 and CD45.2 positive peripheral white blood cells were counted by flow cytometry. The percentage of CD45.1 (host, blue) and CD45.2 (donor, red) cells is shown. Each data point is from a different mouse with bars representing the mean and standard error. B) Mice in A) were treated with tamoxifen and bone marrow isolated 10 days later. RNA was extracted and *Thoc1* RNA levels measured by real time RT-PCR. Each data point is from a different *Thoc1^F/F^*:*Rosa26^CreERT2^* (F/F) or *Thoc1^+/+^*:*Rosa26^CreERT2^* (+/+) mouse, and the data are normalized to one of the control mice. The difference in *Thoc1* RNA levels between genotypes is significant (t-test P = 0.003). C) Protein was extracted from the thymus (1–6) and spleen (7–9) of tamoxifen treated *Thoc1^F/F^*:*Rosa26^CreERT2^* (red) and *Thoc1^+/+^*:*Rosa26^CreERT2^* (blue) mice in B). Extracts were analyzed for the indicated proteins with actin serving as a loading control. D) The small intestine was harvested from mice in B) and tissue sections stained with H&E. Representative images from mice of the indicated genotype are shown. Scale bars represent 200 microns. E) Peripheral blood was isolated from tamoxifen treated *Thoc1^F/F^*:*Rosa26^CreERT2^* (F/F) or *Thoc1^+/+^*:*Rosa26^CreERT2^* (+/+) mice in B) and the percentage of the indicated cell types counted. Each data point is from a different mouse with bars representing the mean. Significant differences (t-test P<0.02) are noted by *. F) Spleen cells were isolated from the tamoxifen treated mice in B) and the percentage of the indicated cell types counted as in E). Significant differences (t-test P<0.01) are noted by ∧. F) Lymph node cells were isolated from the tamoxifen treated mice in B) and the percentage of the indicated cell types counted as in E).

To overcome the potential bias of non-cell autonomous effects, we reconstituted irradiated wild type mice with bone marrow from untreated *Thoc1^F/F^*:*Rosa26^CreERT2^* or *Thoc1^+/+^*:*Rosa26^CreERT2^* mice. Since untreated mice retain *Thoc1*, bone marrow from both genotypes was expected to successfully reconstitute irradiated host mice. Indeed, nine weeks after irradiation and reconstitution more than 85% of white blood cells in the peripheral blood of host CD45.1 expressing mice were derived from the CD45.2 expressing donor bone marrow, on average (Fig. 2A). Reconstituted mice were treated with tamoxifen and RNA or protein extracts prepared 10 days after the start of treatment. As expected, tamoxifen treatment significantly reduced *Thoc1* RNA levels in the bone marrow of *Thoc1^F/F^*:*Rosa26^CreERT2^* reconstituted mice relative to *Thoc1^+/+^*:*Rosa26^CreERT2^* control mice (Fig. 2B, t-test P = 0.003). *Thoc1* protein levels were reduced in secondary hematopoietic organs like the thymus and spleen (Fig. 2C). Consistent with prior studies [25], *Thoc5* protein levels also declined in *Thoc1* deficient tissues (Fig. 2C), suggesting the entire THO complex becomes unstable when one subunit is missing. The small intestine of reconstituted mice was not detectably affected by tamoxifen treatment (Fig. 2D), as expected, since floxed *Thoc1* alleles are now present only in the hematopoietic system. Thus tamoxifen treatment of mice reconstituted with *Thoc1^F/F^*:*Rosa26^CreERT2^* bone marrow caused *Thoc1* deletion specifically in the hematopoietic system.

Peripheral blood, spleen, and lymph node tissues isolated from tamoxifen treated, reconstituted mice were analyzed for various hematopoietic cell types. The relative proportion of *CD11b^+^Gr−1^+^* neutrophils was significantly decreased in each of these tissues (Fig. 2E–G). The decrease in peripheral blood neutrophils was in contrast to the increase observed upon global *Thoc1* deletion, confirming the influence of non-cell autonomous effects on hematopoiesis under inflammatory conditions associated with small intestinal damage. A statistically significant decrease in *CD11b^+^GR−1^negative^* monocytes was also observed in the spleen (Fig. 2F). Significant changes in the proportion of *CD4^+^* cells, *CD8^+^* cells, or *B220^+^* B cells were not observed within the tissues examined, other than a modest increase in peripheral blood B cells (Fig. 2E). The consistent decrease in neutrophils in tamoxifen treated mice reconstituted with *Thoc1^F/F^*:*Rosa26^CreERT2^* bone marrow suggests that the myeloid lineage is sensitive to *Thoc1* deficiency under the conditions tested.

**Figure 3:**
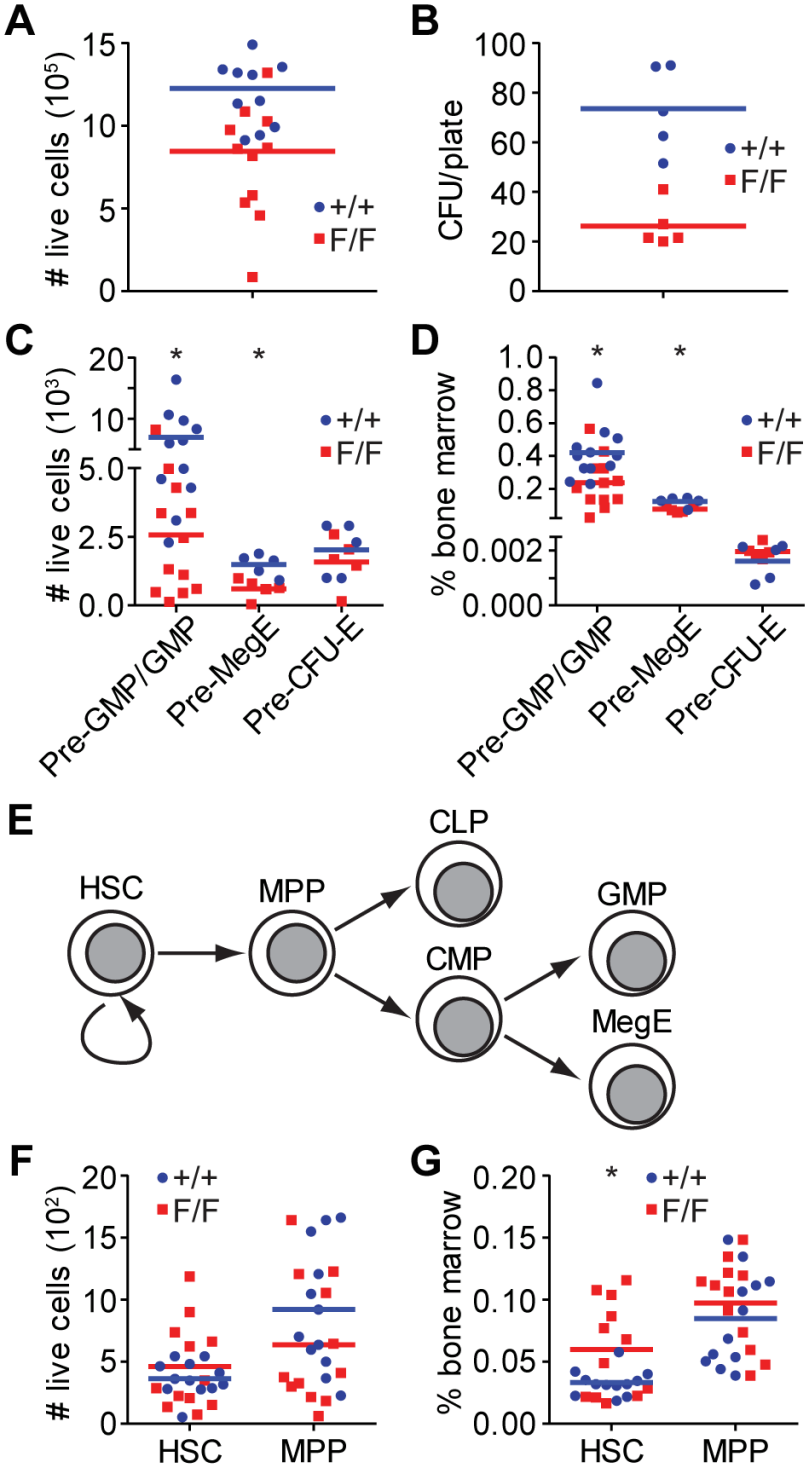
Myeloid progenitor cells are affected by Thoc1 deficiency. A) Bone marrow was recovered from tamoxifen treated *Thoc1^F/F^*:*Rosa26^CreERT2^* (F/F) or *Thoc1^+/+^*:*Rosa26^CreERT2^* (+/+) mice and the number of viable cells counted by flow cytometry. Each data point is from a different mouse with bars representing the genotype mean. Differences between genotypes are significant (t-test P = 0.01). B) An equal number of viable bone marrow cells isolated in A) were cultured in methylcellulose to assess colony forming potential. Each data point shows the number of colonies generated with samples from a different mouse with bars representing the mean. Differences between genotypes are significant (t-test P = 0.0006). C) Pre-granulocyte macrophage progenitors (Pre-GMP), granulocyte macrophage progenitors (GMP), pre-megakaryocyte erythroid progenitors (Pre-MegE), and erythroid progenitors (Pre-CFU-E) were counted in the bone marrow from A) using immunophenotyping and flow cytometry. Each data point is from a different mouse with bars representing the mean. Significant differences (t-test P<0.01) between genotypes are noted by *. D) Data from C) is plotted as a percentage of total viable bone marrow cells analyzed. Significant differences (t-test P<0.02) between genotypes are noted by *. E) A schematic outlining a simplified view of hematopoiesis highlighting the bifurcation of multi-potent progenitor cells (MPP) cells into common lymphoid progenitor cells (CLP) or common myeloid progenitor cells (CMP). HSC indicates hematopoietic stem cells. F) HSC and MPP cells from bone marrow in A) were counted as in C). Results from the different genotypes are not significantly different (t-test P>0.18). G) Data from E) is plotted as a percentage of total viable bone marrow cells analyzed. Significant differences (t-test P<0.05) between genotypes are marked by *.

Myelopoiesis was assessed in tamoxifen treated reconstituted mice by examining the bone marrow. The number of viable bone marrow cells recovered from treated *Thoc1^F/F^*:*Rosa26^CreERT2^* mice was significantly lower than the number recovered from treated *Thoc1^+/+^*:*Rosa26^CreERT2^* control mice (Fig. 3A). Further, viable bone marrow cells recovered from tamoxifen treated *Thoc1^F/F^*:*Rosa26^CreERT2^* mice were less able to form colonies upon *in vitro* culture in methylcellulose (Fig. 3B). Thus, the number and proliferative potential of bone marrow progenitors is reduced upon *Thoc1* loss. Since cells of the granulocyte and macrophage lineages normally make up the majority of bone marrow cells, this data was an initial indicator that granulocyte-macrophage progenitors (GMPs) may be affected by *Thoc1* deficiency. Lineage specific markers were used to quantitate the number of myeloid progenitor cells in the bone marrow to test this possibility. The number and proportion of Pre-GMP and GMP cells was significantly reduced in *Thoc1* deficient bone marrow (Fig. 3C,D). Pre-megakaryocyte erythroid progenitor (Pre-MegE) cells were also reduced in *Thoc1* deficient bone marrow, but this did not significantly affect the number of erythroid-committed-progenitor cells (Pre-CFU-E) at the time points examined (Fig. 3C,D).

The myeloid and lymphocyte lineages bifurcate when multi-potent progenitor cells (MPP) differentiate into a common myeloid or a common lymphoid progenitor cell (Fig. 3E). We have examined whether *CD150^+^* hematopoietic stem cells (HSC) and *CD150^−^* MPP cells that precede the myeloid/lymphoid bifurcation are affected by *Thoc1* loss. The number of HSC or MPP cells was similar in tamoxifen treated *Thoc1^F/F^*:*Rosa26^CreERT2^* and *Thoc1^+/+^*:*Rosa26^CreERT2^* mice (Fig. 3F). The relative proportion of hematopoietic stem cells increased significantly (Fig. 3G), presumably due to the significant loss of myeloid progenitors noted above. Loss of myeloid progenitors likely explains the reduced number of neutrophils and monocytes observed in secondary hematopoietic tissues like the spleen. These observations confirm that the myeloid lineage is preferentially and adversely affected by *Thoc1* deficiency under the conditions tested.

**Figure 4:**
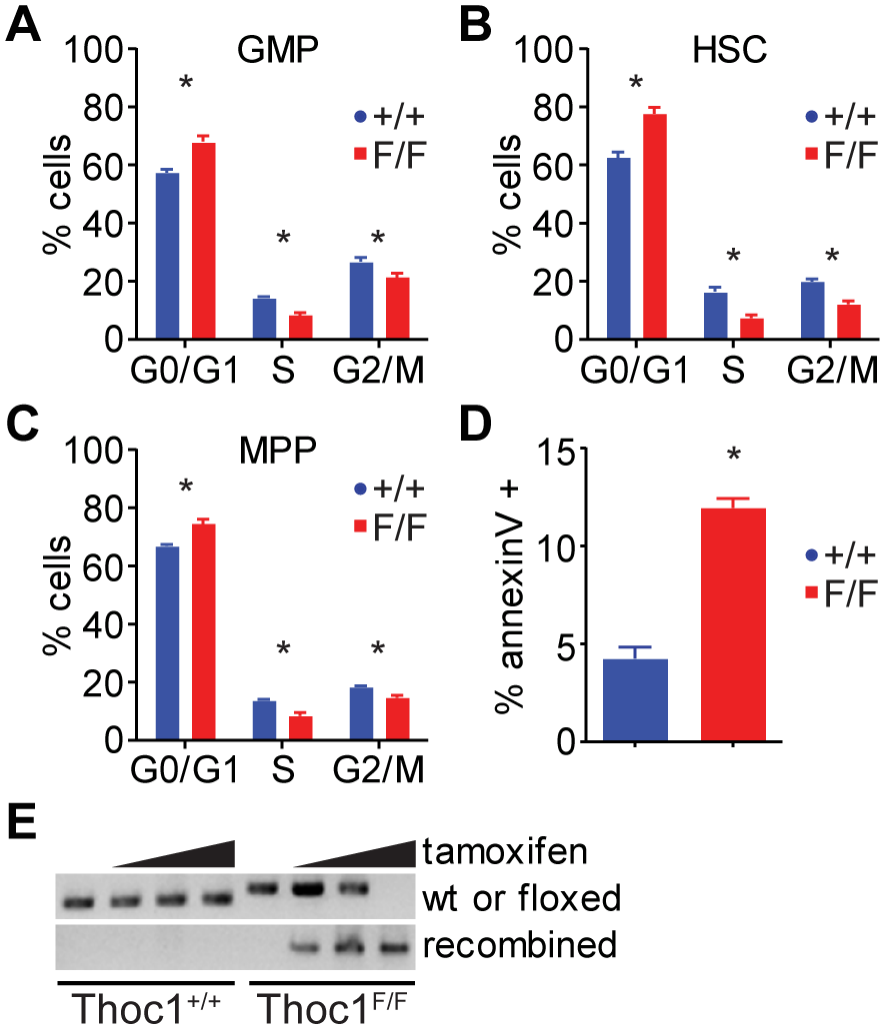
Effects of Thoc1 deficiency on myeloid progenitor cell cycle and apoptosis. A) The cell cycle phase distribution of bone marrow GMPs from tamoxifen treated mice of the indicated genotype was measured by Hoechst 33343 staining and flow cytometry. The graph shows the mean and standard error for 7 different mice of each genotype. Asterisks mark statistically significant differences between genotypes (t-test P<0.01). B) The cell cycle distribution of HSC cells from tamoxifen treated mice was determined. Asterisks mark statistically significant differences between genotypes (t-test P<0.01). C) The cell cycle distribution of MPP cells from tamoxifen treated mice was determined. Asterisks mark statistically significant differences between genotypes (t-test P<0.01). D) The graph shows the percentage of in vitro cultured GMPs of the indicated genotype that stain positive for the apoptotic marker Annexin V and negative for DAPI subsequent to tamoxifen treatment. The data show the mean and standard error for a total of 7 different mice for each genotype analyzed in 2 independent pools of samples. Asterisks mark statistically significant differences between genotypes (t-test P<0.01). E) GMPs in D) were monitored for Cre mediated deletion of the floxed *Thoc1* allele by PCR and agarose gel electrophoresis. GMPs of the indicated genotype were treated with increasing doses of tamoxifen. The upper panel shows results from PCR using primers specific for the wild type or unrecombined floxed *Thoc1* alleles. The lower panel shows results using primers specific for the Cre deleted floxed *Thoc1* allele.

The decrease in myeloid progenitor cells could be due to an inability to proliferate, a loss in viability, or a change in the kinetics of myeloid differentiation. The cell cycle phase distribution of pre-GMP/GMP cells from tamoxifen treated *Thoc1^F/F^*:*Rosa26^CreERT2^* mice showed a significant increase in G0/G1 cells and a concomitant decrease in the proportion of cells in S or G2/M compared to similarly treated *Thoc1^+/+^*:*Rosa26^CreERT2^* mice (Fig. 4A). However, similar changes were also observed in the cell cycle distribution of HSCs and MPPs (Fig. 4B,C). While these changes are consistent with results from the colony formation assay and suggest *Thoc1* deficiency causes decreased cell proliferation, they do not account for the preferential loss of pre-GMP/GMP cells observed upon *Thoc1* loss in vivo. Further, since all bone marrow cells lose *Thoc1* subsequent to tamoxifen treatment, we cannot exclude the possibility that non-cell autonomous effects contribute to the loss of pre-GMP/GMP cells. To address these issues, we isolated pre-GMP/GMPs from the bone marrow of *Thoc1^F/F^*:*Rosa26^CreERT2^* and *Thoc1^+/+^*:*Rosa26^CreERT2^* mice and treated them with tamoxifen *ex vivo*. Tamoxifen treatment caused an increase in the percentage of apoptotic *Thoc1^F/F^*:*Rosa26^CreERT2^* pre-GMP/GMPs, as indicated by annexin V staining, compared to similarly treated *Thoc1^+/+^*:*Rosa26^CreERT2^* cells (Fig. 4D). Increased annexin V staining was accompanied by efficient deletion of the floxed *Thoc1* alleles (Fig. 4E). Hence pre-GMP/GMPs have a cell autonomous requirement for *Thoc1* to maintain cell viability.

## Discussion

The data presented indicate that *Thoc1* deficiency can have both cell autonomous and non-cell autonomous effects on hematopoiesis in the adult mouse, and the effects observed are dependent on cell lineage. The myeloid lineage clearly has a cell autonomous requirement for *Thoc1* as demonstrated by reduced neutrophil numbers in the peripheral blood, spleen, and lymph nodes following hematopoietic-specific *Thoc1* deletion. Both granulocyte-macrophage progenitors and megakaryocyte-erythroid progenitors are reduced in the bone marrow, and granulocyte-macrophage progenitors require *Thoc1* to maintain cell viability when cultured in vitro. Whole body *Thoc1* deficiency, however, increases neutrophils numbers in the peripheral blood. This is likely a non-cell autonomous consequence of increased neutrophil mobilization that is triggered by bacterial infections resulting from loss of epithelial integrity in the gut.

In contrast to the myeloid lineage, lymphocytes are not significantly affected by *Thoc1* loss under the conditions tested. Lymphocyte numbers in the peripheral blood, spleen, and lymph nodes remain similar to wild type control mice retaining *Thoc1*. The only significant difference noted is an increase in the relative percentage of B cells in peripheral blood. While this increase in B cells highlights the different effects of *Thoc1* deficiency on myeloid and lymphoid lineages, the cause is unclear. One possibility is that *Thoc1* deficiency alters the rate of class switch recombination. *Thoc1* deficiency can cause increased accumulation of R-loops, and R-loops are an intermediate in class switch recombination [31]. Increased R-loop formation in *Thoc1* deficient B-cells may increase class-switch recombination and the generation of plasma cells migrating to the peripheral blood. As expected based on the differential effects on the myeloid and lymphoid lineages, the number of HSC and MPP cells which precede myeloid/lymphoid commitment are also largely unaffected by *Thoc1* loss. While lymphocytes are minimally affected, it is tempting to speculate that *Thoc1* loss may have non-cell autonomous effects on adaptive immunity. Adaptive immunity during an antigenic challenge may be compromised indirectly by adverse effects of *Thoc1* deficiency on myeloid-derived dendritic cells. It is also possible that *Thoc1* loss would affect lymphocytes if examined over longer periods of time.

Our results are generally consistent with those observed after inducible deletion of *Thoc5*, another component of the THO complex [25], [32], [33]. Deleting *Thoc5* has deleterious effects on the gut and myeloid lineages of the hematopoietic system. In contrast to our observations, deleting *Thoc5* reduces red blood cell and platelet counts in the peripheral blood. This difference may be explained by the distinct expression pattern of Cre transgenes used in the studies, the known side-effects of interferon treatment on hematopoiesis, the time course of the experiments, or distinct functions for the *Thoc1* and *Thoc5* encoded proteins. This latter possibility seems unlikely in light of the observation that *Thoc5* protein levels decline in *Thoc1* knockout tissue and vice versa [25]. This suggests that THO complex subunits become unstable when the complex does not assemble normally [34].

Lineage dependent effects of THO complex deficiency have now been observed in multiple biological contexts [25], [27], [35], [36]. The biological basis for the specificity of these effects is not currently understood. One possible explanation is that the THO complex regulates a subset of transcripts important for the cell types affected. For example, the THO complex may regulate a subset of transcripts uniquely important for the differentiation, proliferation or viability of myeloid cells thus rendering myeloid cells uniquely sensitive to *Thoc1* loss. Consistent with this hypothesis, *Thoc5* deficient myeloid cells show defects in the expression of some genes that potentially influence myeloid differentiation [32]. Gene expression profiling of other THO deficient cell types has detected changes in different subsets of transcripts [21], [35], [37], [38], suggesting the transcripts regulated by THO vary by cell type. How THO selects transcripts for regulation is unknown, but elucidation of these mechanisms will be required to validate this hypothesis.

Alternatively, THO may regulate a large number of gene transcripts in most cells. Chromatin immunoprecipitation data from yeast indicates that the THO complex regulates the transcripts for most actively transcribed genes [20]. If this extends to mammalian cells, *Thoc1* deficiency would be expected to cause a general decline in the efficiency of gene expression. In this case, differential effects of THO deficiency on individual transcripts is expected to be modest, as is typically observed [21], [32], [35], [37], [38], and highly expressed genes would likely show the greatest defects [20]. Widespread defects in gene transcription may also account for the accumulation of R-loops and DNA damage that is observed in THO deficient cells [31]. In this scenario, cell types that turn over rapidly would be expected to suffer the most from *Thoc1* loss. Rapid cell turnover requires high levels of gene expression to meet the demands of cell growth. Further, rapidly proliferating cells replicate their DNA frequently, making them particularly susceptible to DNA damage that is generated when replication forks collide with R-loops [31]. Indeed one characteristic shared by the cell types acutely sensitive to *Thoc1* loss is their relatively rapid turnover. The epithelium of the small intestine completely turns over every 3–5 days in the mouse [39], while 1×10^9^ neutrophils are produced per kilogram per day in humans [40]. This alternative hypothesis predicts that *Thoc1* loss would also affect lymphocytes if examined under conditions like adaptive immunity where transcriptional programs drive robust lymphocyte proliferation and production of effector molecules. More study is required to test these different and mutually exclusive hypotheses.

The role that RNP complexes play in supporting coordinated gene expression programs is increasingly appreciated [3], [4], but the requirements for individual complexes in normal development and homeostasis have not been explored extensively. The results described here demonstrate that myeloid progenitor cells have a cell intrinsic requirement for the *Thoc1* encoded protein, an essential component of the THO RNP complex. The effects of *Thoc1* deficiency in the hematopoietic system are lineage dependent. While we cannot exclude the possibility that resistant lineages like lymphocytes would be affected by *Thoc1* loss under different conditions, these observations indicate *Thoc1* and THO complex deficiency have context dependent effects on hematopoiesis in the adult mouse. Context dependent effects have also been observed in other tissues like the intestine [27], [33], suggesting this may be a general theme. Elucidation of the mechanisms underlying these context dependent effects will inform our understanding of how RNP complexes contribute to normal development, tissue homeostasis, and disease.

## Supporting Information

Table S1 (DOCX)
