# Supplementary material for: The Thoc1 Encoded Ribonucleoprotein Is Required for Myeloid Progenitor Cell Homeostasis in the Adult Mouse
Source: PLoS One. 2014 May 15;9(5):e97628. doi: 10.1371/journal.pone.0097628 (PMC4022742; doi:10.1371/journal.pone.0097628)
Supplement: Table S1 — (DOCX) [file pone.0097628.s001.docx]

Supplementary Table S1: Antigens used for lineage marking.

| Antigen | Fluorochrome | LSK cells | HSC | MPP | Pre GM | GMP | PreMegE | CFU-E |
| --- | --- | --- | --- | --- | --- | --- | --- | --- |
| Lineage | FITC | - | - | - | - | - | - | - |
| Sca-1 | PE | + | + | + | - | - | - | - |
| c-kit | APC-eflour 780 | + | + | + | + | + | + | + |
| CD150 | PE cy7 |  | + | - | - |  | + | - |
| CD16/32 | PerCpCy5.5 |  |  |  |  | + |  |  |
| CD105 | Pacific blue |  |  |  | - |  | - | + |
| Live/Dead | DAPI |  |  |  |  |  |  |  |
